# Prenatal Trimethyltin Exposure Induces Long-Term DNA Methylation Changes in the Male Mouse Hippocampus

**DOI:** 10.3390/ijms22158009

**Published:** 2021-07-27

**Authors:** Soon-Ae Kim, Jung-Hoon Chai, Eun-Hye Jang

**Affiliations:** 1 Department of Pharmacology, School of Medicine, Eulji University, Daejeon 34824, Korea; dmter12@gmail.com; 2 Center for Sport Science in Seoul, Seoul Sports Council, Seoul 02119, Korea; jhchai80@gmail.com

**Keywords:** trimethyltin, prenatal, mitochondria, epigenetic, FOXO3

## Abstract

Trimethyltin (TMT) is an irreversible neurotoxicant. Because prenatal TMT exposure has been reported to induce behavioral changes, this study was conducted to observe gender differences and epigenetic changes using a mouse model. In behavioral testing of offspring at 5 weeks of age, the total times spent in the center, corner, or border zones in the male prenatal TMT-exposed mice were less than those of control unexposed mice in the open-field test. Female TMT-exposed mice scored lower on total numbers of arm entries and percentages of alternations than controls in the Y-maze test with lower body weight. We found that only TMT-exposed males had fewer copies of mtDNA in the hippocampus and prefrontal cortex region than controls. Additional epigenetic changes, including increased 5-methyl cytosine/5-hydroxymethyl cytosine levels in the male TMT hippocampus, were observed. After methylation binding domain (MBD) sequencing, multiple signaling pathways related to metabolism and neurodevelopment, including FoxO signaling, were identified by pathway analysis for differentially methylated regions (DMRs). Increased FOXO3 and decreased ASCL1 expression were also observed in male TMT hippocampi. This study suggests that sex differences and epigenetics should be more carefully considered in prenatal toxicology studies.

## 1. Introduction

Trimethyltin (TMT) accumulates in the body after acute exposure [1]. Stannin (Snn) is a highly conserved 88-residue protein that is localized to mitochondria and may mediate the selective toxicity of TMT [2]. TMT exhibits neurotoxic effects that are localized in the limbic system, particularly in the hippocampus, in both experimental animals and accidentally exposed humans [3,4]. Behavioral tests have demonstrated that TMT exposure damages the hippocampus, resulting in memory and learning deficits [5,6]. TMT-exposed rats showed postnatal toxicity and decreased birth weights [7]. Several animal models involving prenatal chemical exposure to substances including valproic acid and TMT for neurodevelopmental disorders have been suggested [8,9]. Others have reported that several human neurodevelopmental disorders, including autism spectrum disorder and attention deficit hyperactivity disorder, show changes in peripheral mitochondrial DNA copy number, which may be related to mitochondrial dysfunction [10,11,12].

Furthermore, it is suggested that in utero environmental stress exposure could result in long-term epigenetic alterations, which induce consequences for development in the offspring [13]. While there are multiple modes of epigenetic modulation, DNA methylation is the most heavily studied and there is only limited work considering the effects of prenatal substance exposure on differential microRNA or histone modifications. In addition, depletion of mtDNA has been reported to cause significant changes in the methylation patterns of multiple genes, providing the first direct evidence that mitochondria regulate epigenetic modifications in the nucleus [14]. Several studies have identified possible effects on reversible and irreversible changes in genomic DNA methylation profiles of the nuclear genome as a consequence of mitochondrial dysfunction [15]. It may be possible that prenatal exposure to TMT induces epigenetic and signal transduction changes during long-term neurodevelopment. However, sex differences in behavior and phenotypes were not considered. Therefore, this study aimed to evaluate sex-specific behavioral effects and the possibility of epigenetic modification of genes involved in nervous system development by TMT exposure during the prenatal period.

## 2. Results

### 2.1. Prenatal TMT Exposure Induces Sex-Specific Behavioral Changes

Open-field testing showed no significant difference in distances traveled between the TMT-treated group (TMT, n = 16) and the control (CON, n = 17) groups when males and females were combined. The TMT group spent significantly less time in the center (*p* = 0.015) and around the borders (*p* = 0.001), and significantly more time in the corners than the CON group (*p* = 0.01). The data were reanalyzed for sex-specific differences. There was no significant difference in the distance traveled between TMT and (n = 8) and CON (n = 10) males. TMT males spent significantly less time in the center (*p* = 0.008) and around the borders (*p* = 0.013), with significantly more time in the corners than CON males (*p* = 0.002). On the other hand, the distances traveled by TMT females were significantly less than those of the CON group (*p* = 0.049), but there were no differences in time spent in the center, along the borders, or the corners. In addition, the total numbers of alternations and the percentages of alternations per trial were not significantly different between TMT and CON groups in the Y-maze test. However, the total number of arm entries (*p* = 0.036) and the number of alternations (*p* = 0.003) exhibited by TMT females were less than those for CON females (Table 1). Two-way ANOVA confirmed that gender-dependent interactions occurred only for the time of stay in the corner of the open-field test (Table S1).

Body weight was analyzed according to sex and prenatal TMT exposure at 6 weeks old. Although there were no differences in body weights between males in the TMT group (19.9 ± 1.7 g) and males in the CON group (20.8 ± 1.9 g), females in the TMT group (16.4 ± 0.9 g) had significantly lower body weight than females in the CON group (17.9 ± 1.1 g) (*p* = 0.004). The mean body weight of the TMT group (18.3 ± 2.2 g) was significantly lower than that of the CON group (19.7 ± 2.1 g) (*p* = 0.049).

### 2.2. Decreased Mitochondrial DNA Copy Numbers in Male Hippocampus and Prefrontal Cortex

The mtDNA copy number found in the hippocampus and prefrontal cortex of males in the TMT group (n = 13) was smaller than that of males in the CON group (n = 13), but there was no significant difference in the cerebellum. No significant differences in mtDNA copy number were found in the hippocampus, cerebellum, or prefrontal cortex between CON and TMT groups of female (Table 2). If sex was not considered, the mtDNA copy number in the hippocampus was significantly lower in the TMT group (n = 23) than in the CON group (n = 21) (*p* = 0.049), but there were no significant differences between groups in the cerebellum and prefrontal cortex. We also observed correlations between mtDNA and behavior data from OFT and Y-maze with tissue and sex specificity (Tables S1 and S2). 

### 2.3. DNA Methylation Levels and DNMT1 mRNA Expression Levels Are Altered in the Prenatal TMT-Exposed Male Hippocampus

**Figure 1:**
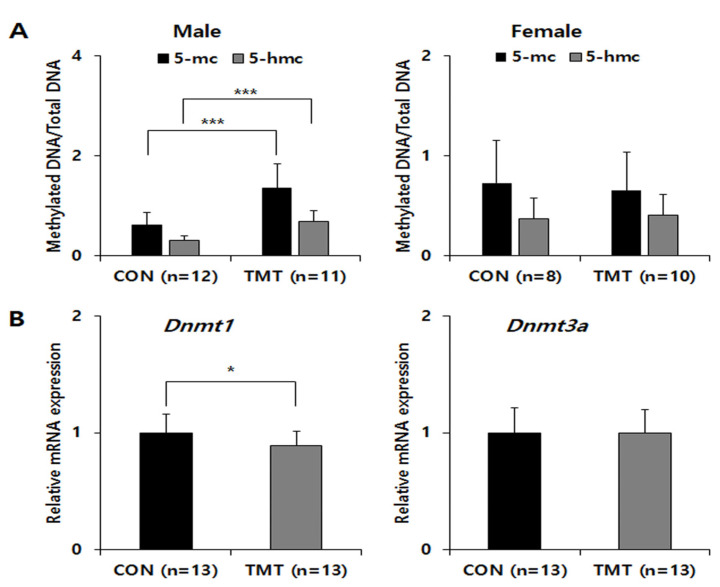
Global DNA methylation and Dnmt gene expression in the hippocampi of mice prenatal exposed to TMT. (**A**) Sex differences in global cytosine methylation and hydroxymethylation in genomic DNA samples of control and TMT groups. (**B**) Relative mRNA expression of *Dnmt1* and *Dnmt3a*, normalized to *Gapdh* as an internal control in the male hippocampus. * *p* < 0.05, *** *p* < 0.001 compared to control (CON). Data are shown as means ± standard deviations (SD).

**Figure 2:**
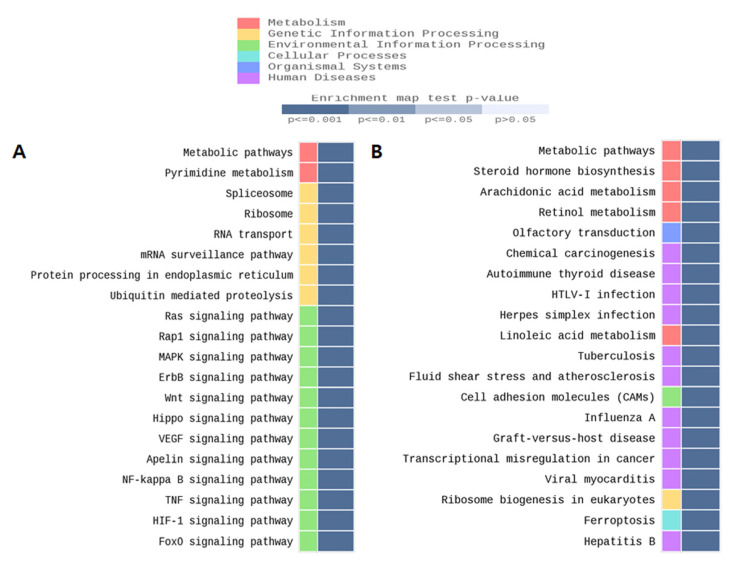
Top-ranked gene enrichment networks from querying the KEGG database with MBD-seq data from prenatal TMT exposed male hippocampus. (**A**) CGI promoter region. (**B**) Non-CGI promoter region.

Global DNA methylation levels were not statistically significant in females, but a significant increase was observed in the male TMT group only (Figure 1A). The 5-methylcytosine % (mean ± standard deviation) was 1.35 ± 0.50 in the TMT group compared with 0.60 ± 0.26 the CON group (*p* ≤ 0.001). The 5-hydroxymethylcytosine (mean% ± standard deviation) was 0.679 ± 0.23 in the TMT group compared with 0.30 ± 0.10 in the CON group (p < 0.001). To analyze Dnmt gene expression changes, we performed qPCR for *Dnmt1* and *Dnmt3a* on male hippocampal RNA (Figure 1B). Although the relative mRNA expression of *Dnmt3a* was not different between groups in males, the expression of *Dnmt1* mRNA was significantly decreased in the TMT prenatal male hippocampus (*p* = 0.048). We performed MBD-sequencing to select candidate genomic regions of DNA methylation change by prenatal TMT exposure with pooled genomic DNA extracted from each male hippocampus, since only male samples show a statistically significant increase of DNA methylation level. After genome-wide distribution of DMR analysis with fold change and statistical test, we identified 2809 hypermethylated regions and 2778 hypomethylated regions in the CGI promoter. In addition, 2009 hypermethylated regions and 2183 hypomethylated regions were identified in the non-CGI promoter (Table S3). After gene network analysis of DMRs in promoters and exons, we observed several significant enrichments for several gene ontology (GO) terms and KEGG/Reactome pathways for both groups, including the metabolic and FOXO signaling pathways (Figure 2).

### 2.4. Prenatal TMT Exposure Induces Changes in the Expression of Mitochondria-Related Genes and FOXO3 in the Male Hippocampus

**Figure 3:**
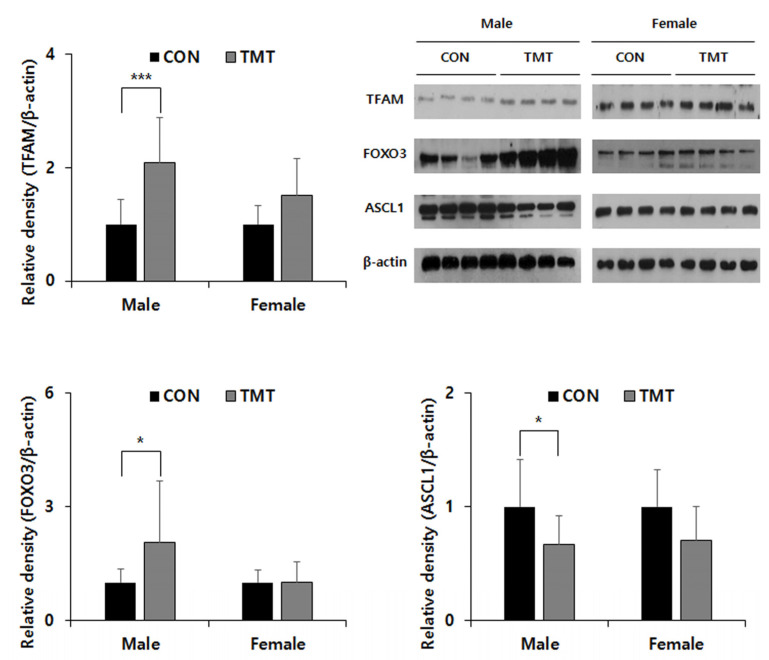
Protein expression of TFAM, FOXO3 and ASCL1 in the VPA exposed hippocampus. FOXO3, Forkhead box O3a; TFAM, Transcription Factor A, Mitochondrial; ASCL1, Achaete-Scute Family BHLH Transcription Factor 1. * *p* < 0.05, *** *p* < 0.001 compared to control (CON). Data are shown as means ± standard deviations (SD).

To confirm the reliability of the GO analysis of the DMR data, we selected the mitochondria-related Tfam gene for the metabolism pathway, Foxo3 gene for the FOXO signaling pathway and the Ascl1 gene was additionally selected and evaluated their mRNA expression by qPCR. Although there was no change in mRNA expression of *Tfam* and *Ascl1*, an increase in the mRNA expression level of Foxo3 gene in TMT male hippocampus (*p* = 0.039) can be observed. In the Western blot analysis (Figure 3), though there was no difference between CON and TMT groups in the female hippocampus, TFAM expression was increased in the TMT-exposed male hippocampus (*p* < 0.001). An increasing FOXO3 expression in male hippocampus exposed to prenatal TMT (*p* = 0.041) was also observed. In addition, we observed a decreased expression of ASCL1, which has been reported to be a crucial regulator of multiple aspects of neurogenesis and shares common targets with FOXO3 in the male hippocampus (*p* = 0.024).

## 3. Discussion

In this study, we observed the sex-specific effects of prenatal TMT exposure on behavior, suggesting a role in neuropsychiatric disorders. In general, toxicity is observed as weight loss in addition to hyperexcitability, convulsions, and posterior paresis [16]. In most previous studies using the prenatal stress model, only males were selected for study [6,9]. The open-field test, which is most commonly used for animal behavioral testing, was used to evaluate locomotor activity and anxiety [17]. In this study, TMT-exposed males spent less time in the center and along the borders and more time in the corners during the open-field test. These results are suggestive of anxiety, a neurotoxic effect of TMT, and appear predominantly in male mice [18]. However, a limitation of this study is that the sample sizes of both sexes in the behavior tests were dissimilar. Several hypotheses have been proposed to explain sex differences in response to the same environmental exposure during the perinatal period, such as sex hormones and placental protein expression differences, but these suggestions have not been rigorously explored [19]. Therefore, it is necessary to consider sex differences in further studies of animal prenatal stress models. 

We also observed that a single prenatal exposure to the minimum toxic dose of TMT caused a decrease in the mtDNA copy numbers in the hippocampus and prefrontal cortex of males, but not in females. Mitochondria exert multiple functions in cellular metabolism and redox homeostasis and are known as the powerhouses of the cell. They have been increasingly revealed to be crucial for a broad range of neural processes and general brain function. There is increasing evidence of a role for mitochondria in the etiology of neuropsychiatric disorders [20,21]. Oxidative stress caused by mitochondrial dysfunction due to TMT exposure may contribute to behavioral disorders or neurological disease [22,23]. It has also been suggested that brain mitochondrial dysfunction may be associated with behavioral abnormalities including anxiety and depressive-like behavior. Several animal studies have reported anxiety-like behaviors and physiological responses to stress change accompanied by variations in mtDNA [24]. Mitochondrial biogenesis, which responds to cellular energy following starvation or oxidative stress, is indirectly measured by mtDNA copy number. Decreased mtDNA copy numbers at six weeks may represent brain mitochondrial dysfunction and oxidative stress [25]. Several studies have consistently shown alterations in mtDNA copy number in peripheral samples from psychiatric patients with neurodevelopmental disorders such as ASD (Autism Spectrum Disorder) [11]. Although we observed that the expression level of *Tfam* was increased in the male hippocampus, the mtDNA copy number was decreased. Since the mtDNA copy number can be changed by the expression of multiple genes including *Tfam*, complex etiology such as epigenetic factors can be inferred, but this study could not clearly elucidate them.

Multiple prenatal factors affect neurodevelopment. Similarly, several prenatal chemical injection animal models have been used to study neuropsychiatric defects. For example, nicotine is the most common substance used during pregnancy, with ADHD (Attention Deficit Hyperactivity Disorder) known to be more prevalent in children exposed prenatally to tobacco [26]. This may be a better representation of the environmental/epigenetic factors than genetic factors using transgenic models with mutations in single risk genes [27]. In particular, in utero exposure to VPA in rodents has been used as a model for ASD with predictive validity [28]. In addition, several prenatal immune challenge models have been introduced in the case of schizophrenia, which shares the symptoms of ASD [29]. Epigenetic mechanisms, such as DNA methylation and histone modification, may be key factors in explaining these neurodevelopmental animal models. Increased DNA methylation was observed only in the male hippocampus in this study. It has also been suggested that FOXO signaling can be affected by DNA methylation through gene network analysis by MBD-seq and qPCR. Mammals have four FOXO proteins, 1, 3, 4, and 6, which show a high sequence similarity. It has been suggested that the activation of FOXO3, which is mainly expressed in mitochondria, can induce specific sets of nuclear genes, including cell-cycle inhibitors, pro-apoptotic genes, reactive oxygen species (ROS) scavengers, autophagy effectors, gluconeogenic enzymes, and others depending on context [30]. In addition, several groups have reported that FOXO3 activates not only the repression of a large number of nuclear-encoded genes with mitochondrial function, but also inhibits pro-neuronal bHLH transcription factor ASCL1-dependent neurogenesis [31]. In addition, it can be considered that prenatal substance exposure may share other epigenetic mechanisms such as histone modification, and microRNA, since there were gender differences in FOXO3 gene expression levels in this study. Furthermore, although there was no change in mRNA expression, increased expression of TFAM and ASCL1 was observed in this study. Therefore, the possibility that the increase expression of TFAM and ASCL1 was caused by post-transcriptional post-translational regulation cannot be excluded.

Multiple studies have implicated disruption of signaling in neurodevelopmental disorders [32,33]. Therefore, exposure to TMT during the developmental phase may induce neurodevelopmental abnormalities through changes in signal transduction. Microarray-based, genome-wide expression analysis has been used to investigate the molecular changes occurring in the TMT-injured brain, suggesting a critical role for mitochondrial dysfunction and disruption of calcium homeostasis in the early phase of TMT-induced neurotoxicity [34]. It is suggested that there is a possible role of Snn in several key signaling systems, including activation of the p38-ERK cascade, p53-dependent pathways, and 14-3-3x protein-mediated processes [35]. Moreover, it may be possible that the difference of *Snn* expression level by sex or substance exposure may affect signaling changes. However, we cannot observe any difference in *Snn* mRNA expression between sex and TMT exposure (data not shown). TMT-treated isolated mitochondria showed a time-dependent inhibition of ADP-stimulated oxygen consumption using succinate or glutamate/malate as substrates [36]. Mitochondrial dysfunction can cause cytotoxicity and apoptosis, including activation of the caspase8/caspase 3 pathway in primary cultured neuronal cells after TMT exposure [37]. Several signaling pathways that have been previously studied in TMT neurotoxicity have also been identified in gene network analyses. For example, Kim et al. reported the involvement of the GSK-3/β-catenin signaling pathway in TMT-induced hippocampal cell degeneration and dysfunction [38]. Moreover, Qing et al. reported that crosstalk between nuclear factor (NF-κB) and mitogen-activated protein kinase (MAPK) may be involved in TMT-induced apoptosis [39].

## 4. Materials and Methods

### 4.1. Animal Model

Seven-week-old male and female mice (C57BL/6) were purchased from Samtako Osan (Korea). In a one-week familiarization, mice were maintained at a temperature of 22 ± 2 °C, under a 12-h light/dark cycle (on 7:00 to 19:00) at 50 ± 10% relative humidity at with food and tap water available ad libitum. After environmental adaptation, 2 female mice and 1 male mouse were mated in one cage. After the pregnancy was confirmed, female mice were placed in each individual cage. We confirmed that the gestation period under these conditions was 22 ± 1 days. Pregnant C57BL/6 mice were once intraperitoneal injected with TMT (2.3 mg/kg) in saline (10 µL/g) or the same volume of saline alone (CON) 12.5 days after mating. At birth, newborns was counted and natural breastfeeding was maintained for approximately three weeks. Males and females were segregated after four weeks. There were more than 4 pregnant mice per group, and the survival days of the offspring was the same, although not all became pregnant at the same time. None of the offspring died shortly after birth or before sacrifice, all male and female mice within a same litter were used in the study. A total of 44 male (n = 26) and female (n = 18) mice were assigned to CON and TMT groups. Behavior tests were conducted at 5 weeks of age. Animals were sacrificed by inhalation anesthesia at six weeks after birth and samples were taken from the hippocampus, cerebellum, and prefrontal cortex of the brain. This study was approved by the Institutional Animal Care and Use Committee of Eulji University (EUIACUC 17-12).

### 4.2. Behavioral Testing

All behavioral experiments were conducted between 9:00 am and 12:00 pm in consideration of murine circadian rhythms (at 5 weeks of age). Open-field tests were performed in a soundproof experimental room using a white open field (the dimensions of 30 × 30 cm ^2^, walls 40 cm high). Initially, mice were gently placed in the center of the arena and allowed to explore. Motor activity was assessed in 5-min sessions, recorded using a video camera (LG, LS903N-B, Seoul, Korea), and scored using video tracking software (Noldus EthoVision, Wageningen, The Netherlands). Total distances and times spent in different zones of the field were recorded and analyzed. The Y-maze was a closed three-arm maze with equal angles between all arms 30 cm long, 5 cm wide, and 12 cm high. Mice were placed at the center of the arms and allowed to move freely through the maze during an 8-min test period. The percentage of trials in which mice entered all three arms was recorded as one alternation. This was used as an estimate of short-term memory. Total numbers and series of arm entries were recorded. The number of maximum alternations was therefore the total number of arm entries minus two, and the percentage of alternations was calculated as (actual alternations/maximum alternations) × 100.

### 4.3. Mitochondrial DNA (mtDNA) Copy Number and DNA Methylation Assays

Total genomic DNA was isolated using a DNeasy blood and tissue kit (Qiagen, Hilden, Germany). Mitochondrial DNA copy number was assessed using quantitative real time polymerase chain reactions (qRT-PCR) and calculated by the 2^−ΔΔCT^ method [40], using the equation: mtDNA copy number = 2^−ΔCt^, where ΔCt = Ct_mitochondria_ − Ct_nuclear_. The β-actin (*Actb*) nuclear gene and genes from mtDNA were amplified by qRT-PCR using iQTM SYBR^®^ Green Supermix (Bio-Rad, Hercules, CA, USA) in a CFX96TM Real-Time system (Bio-Rad, Hercules, CA, USA). To prepare PCR samples, 3 µL of genomic DNA (5 ng/µL) was mixed with 2 µL of each primer (10 pmol/µL), and 5 µL of SYBR supermix. A global DNA methylation assay kit (Abcam, Cambridge, UK) was used to quantify the global hippocampal genomic DNA methylation according to the manufacturer’s instructions.

### 4.4. Real-Time Quantitative Polymerase Chain Reaction 

Total RNA was extracted using the miRNeasy Mini Kit (Qiagen, Hilden, Germany). DNA was stored at −80 °C until use. For relative mRNA expression analysis, cDNA was synthesized using an RT^2^ first strand kit (Qiagen, Hilden, Germany) from total RNA and PCR was performed using the same PCR premix and instrument described above. Expression was normalized to Gapdh gene expression and was assessed using the 2^−^^ΔΔCt^ method. Primer sequences and PCR conditions are listed in Table S4.

### 4.5. MDB Sequencing

Methylated DNA was isolated using the MethylMiner Methylated DNA Enrichment Kit (Invitrogen, Carlsbad, CA, USA) according to the manufacturer’s instructions. Briefly, fragmentation of 1ug of pooled genomic DNA from each group was performed using adaptive focused acoustic technology (AFA; Covaris) and captured by MBD proteins, then eluted in a high-salt buffer. DNA in each eluate was precipitated with glycogen, sodium acetate, and ethanol, then resuspended in DNase-free water. This DNA was used to generate libraries following the standard protocols provided with the TruSeq Nano DNA Library Prep Kit (Illumina, San Diego, CA, USA). The eluted DNA was repaired, an Ais ligated to the 3 end, Truseq adapters are then ligated to the fragments. Once ligation had been assessed, the adapter-ligated product was PCR amplified. The final purified product was then quantified by qPCR according to the qPCR Quantification Protocol Guide and qualified using the Agilent Technologies 4200 TapeStation software (Agilent Technologies, Santa Clara, CA, USA). We then sequenced using the HiSeq™ 2500 platform (Illumina, San Diego, CA, USA).

### 4.6. Data Processing and Methylation Profile Calling 

Paired-end sequencing reads (101 bp) generated from MBD sequencing were verified using FastQC (version 0.10.0). Before starting analysis, Trimmomatic (version 0.32) was used to remove adapter sequences and bases with base qualities lower than 3 from the end reads. Using the sliding window trim method, bases that did not qualify for window size 4 and mean quality 15 were removed. Afterwards, reads with a minimum length of 36 bp were removed. 

The cleaned reads were aligned to the human genome (UCSC mm10) using Bowtie (version 1.1.2 parameter set-n2-m1-X 700), allowing up to 2 nucleotide mismatches to the reference genome per seed and returning only uniquely mapped reads. Mapped data (SAM file format) were sorted and indexed using SAMtools (version 0.1.19). PCR duplicates were removed using Picard Mark Duplicates version 1.118.

Analysis of MBD data was performed using the MEDIPS package (version 1.16.0). For each sample, aligned reads were extended in the sequencing direction to a length of W300 nucleotides. The sequencing read coverage of the extended reads was calculated using a genome-wide, 250-bp window size. Subsequently, the resulting coverage profiles (read count, RPKM, and RMS) for each genomic bin were calculated. Each DMR was annotated using the table browser function of the UCSC genome browser. Annotation included gene structures, transcripts, promoter regions (defined as −2 kb upstream of the transcription start site), exons, introns, and CpG islands. 

### 4.7. Identification of Differentially Methylated Regions (DMRs) 

Read counts for each genomic bin were normalized with TMM (trimmed mean of M-value normalization) in edgeR. We applied an exact test to assess the significance of methylation differences between groups using edgeR. Differentially methylated regions (DMRs) were determined by filtering for each region associated with |log2FC| ≥ 1 and exact test *p*-values < 0.05. Hierarchical clustering analysis also was performed using complete linkage and Euclidean distance as a measure of similarity to display the methylation patterns of DMRs, which were satisfied with |log2FC| ≥ 1 and *p*-values < 0.05 at least one more comparison pairs exact test. Gene-enrichment and functional annotation analysis for the significant gene list was performed using Gene Ontology (www.geneontology.org/, 26 May 2021) and pathway analysis for the DMR was performed based on the KEGG pathway (http://www.genome.jp/kegg/pathway.html, 26 May 2021). All data analysis and visualization of differentially methylated results were conducted using R 3.0.2 (www.r-project.org, 26 May 2021).

### 4.8. Western Blotting 

Tissues (male hippocampus) were homogenized in RIPA buffer (ATTO, Tokyo, Japan) with proteinase and phosphatase inhibitors (ATTO, Tokyo, Japan). Antibodies against β-actin (Cell Signaling, Danvers, MA USA), FOXO3 (Cell Signaling, Danvers, MA, USA), ASCL1 (Abcam, Cambridge, UK), and TFAM (Millipore, Burlington, MA, USA) were used as primary antibodies. After conventional Western blotting, bands were visualized using the Pierce^TM^ ECL western blotting substrate (Thermo Scientific, Waltham, MA, USA). Protein expression levels were calculated using ImageJ software (NIH, Bethesda, MD, USA).

### 4.9. Statistical Analysis

All data, including qPCR and Western blotting, are expressed as means ± standard deviations. Statistical analysis was performed using SPSS version 20.0 (IBM Co., Armonk, NY, USA). The statistical significance of differences between groups was evaluated using a two-tailed Student’s *t*-test. The Mann–Whitney U-test was used for data that were not normally distributed. In addition, a two-way ANOVA was used to confirm the differences according to gender. Correlations between mtDNA copy numbers and behavioral variables were calculated using Pearson’s correlation analysis. A *p*-value less than 0.05 was considered statistically significant, and significance is denoted in graphs as: * *p* < 0.05, ** *p* < 0.01, and *** *p* < 0.001.

## 5. Conclusions

Our data suggest that prenatal TMT exposure induces epigenetic changes, which may affect neurodevelopmental changes in the male hippocampus by altering mitochondrial and FOXO signaling. There is a limit to determine the precise mechanism of FOXO3 gene expression changes for DNA methylation and the effects for mitochondrial DNA copy number with brain region/sex specificity by prenatal TMT exposure, since only the hippocampal region has been studied and the cause of the gender differences and specific valid target genes which are regulated by DNA methylation was not identified in this study. Therefore, it will need additional studies with further animals and human subjects.

## Figures and Tables

**Table 1:** Behavior test results of 5 week-old mice shows sex differences in prenatal TMT treatment.

Gender	Test	Variables	CON	TMT	*p*-Value
Male	Open field test	Distance	3765.5 ± 891.8	3704.3 ± 1021.3	0.859 ^†^
		Center (s)	48.4 ± 36.2	17.3 ± 8.2	0.008 ^‡^
		Corner (s)	333.5 ± 36.3	410.9 ± 36.8	0.002 ^‡^
		Borders (s)	218.2 ± 31.9	171.7 ± 31.6	0.013 ^‡^
	Y-maze	Total number	38.4 ± 8.5	43.4 ± 14.7	0.398 ^†^
		Alternation	22.9 ± 6.3	24.9 ± 8.6	0.532 ^†^
		Alternation %	0.63 ± 0.09	0.6 ± 0.06	0.654 ^†^
Female	Open field test	Distance	3664.5 ± 641.1	3007.2 ± 520.9	0.049 ^‡^
		Center (s)	25.6 ± 16.5	18.4 ± 11	0.487 ^†^
		Corner (s)	395.6 ± 35.4	413.1 ± 36.6	0.355 ^†^
		Borders (s)	178.8 ± 24	168.5 ± 35.1	0.487 ^†^
	Y-maze	Total number	45.1 ± 6	36.4 ± 8.2	0.036 ^‡^
		Alternation	28.3 ± 3.1	20.1 ± 3.6	0.003 ^‡^
		Alternation %	0.66 ± 0.05	0.6 ± 0.07	0.072 ^†^

Male: CON (n = 10), TMT (n = 8), Female: CON (n = 7), TMT (n = 8), ^†^ Mann–Whitney U-test, ^‡^ Student *t*-test.

**Table 2:** Prenatal TMT exposure alters male mitochondrial DNA copy numbers.

Sex	Tissue	CON	TMT	*p*-Value
Male	Hippocampus	2893.8 ± 687.4	2461.2 ± 869.8	0.045 ^‡^
	Cerebellum	603.8 ± 259.4	752.5 ± 587.2	0.817 ^†^
	Prefrontal cortex	2641.8 ± 660.9	2047.6 ± 750.8	0.043 ^‡^
Female	Hippocampus	2610.6 ± 332.6	2784.8 ± 621.5	0.722 ^‡^
	Cerebellum	433.6 ± 77.5	457.9 ± 193.7	0.657 ^†^
	Prefrontal cortex	2978.3 ± 711.3	3237.6 ± 690.4	0.286 ^†^

CON, control, TMT, prenatal TMT treatment, Males: CON (n = 13), TMT (n = 13); Females: CON (n = 8), TMT (n = 10). ^†^ Mann–Whitney U-test, ^‡^ Student *t*-test.

## Data Availability

Data are contained within the article.

## Supplementary Materials

The following are available online at https://www.mdpi.com/article/10.3390/ijms22158009/s1.
