# Supplementary material for: Prenatal Trimethyltin Exposure Induces Long-Term DNA Methylation Changes in the Male Mouse Hippocampus
Source: Int J Mol Sci. 2021 Jul 27;22(15):8009. doi: 10.3390/ijms22158009 (PMC8348768; doi:10.3390/ijms22158009)
Supplement: Supplementary file 1 [file ijms-22-08009-s001.zip › Table S1.pdf]

Table S1. Two way ANOVA results for TMT treatment, sex and interaction on behavior tests.

Test		TMT		Sex		TMT & Sex	
		F	p-value	F	p-value	F	p-value
Open Field Test	Distance (cm)	1.613	0.214	1.991	0.169	1.111	0.301
	Center (sec)	5.891	0.022	1.859	0.183	2.292	0.141
	Corner (sec)	13.837	0.001	6.351	0.017	5.525	0.026
	Border (sec)	6.696	0.015	3.771	0.062	2.722	0.11
Y-maze	Total number	0.296	0.59	0.001	0.971	3.892	0.058
	Alteration	2.19	0.15	0.023	0.88	5.88	0.022*
	Alteration (%)	2.948	0.097	0.316	0.578	0.665	0.421

Data were analyzed by two-way ANOVA. *Tests the null hypothesis that the error variance of the dependent variable is equal across groups.
