# Supplementary material for: Prenatal Trimethyltin Exposure Induces Long-Term DNA Methylation Changes in the Male Mouse Hippocampus
Source: Int J Mol Sci. 2021 Jul 27;22(15):8009. doi: 10.3390/ijms22158009 (PMC8348768; doi:10.3390/ijms22158009)
Supplement: Supplementary file 1 [file ijms-22-08009-s001.zip › Table S4.pdf]

Table S3. PCR Condition

Study	Gene	Primer sequence		PCR condition
mtDNA copy numbers	mtDNA	F	GCCCATGACCAACATAACTG	95 °C for 10 min, 40 cycles at 95 °C for 15 s, 60 °C for 1 min
		R	CCTTGACGGCTATGTTGATG	
	Actb	F	GATGCCACAGGATTCCATACCTA	
		R	AGCCTAGTCCTTTCTCCATCTAAAG	
mRNA expression	*Dnmt1*	F	GAGAACGGAACACACACTCTCACT	
		R	TATTTGAGTCTGCCATTTCTGCTC	
	Foxo3	F	GCAAGCCGTGTACTGTGGA	
		R	CCTGGTGGAATGCACTGCAGAAGGA	
	Dnmt3a	F	GCCGAATTGTGTCTTGGTGGATGACA	95 °C for 3 min, 40 cycles at 95 °C for 15 s, 58.2 °C for 1 min
		R	CCTGGTGGAATGCACTGCAGAAGGA	
	Snn	F	GCCCCTTAGCGCTTTCCTTA	95 °C for 3 min, 40 cycles at 95 °C for 15 s, 55 °C for 1 min
		R	ACGTGCTTCTGGGCCTATTT	
	Gapdh	F	GAGTCAACGGATTTGGTCGT	95 °C for 3 min, 40 cycles at 95 °C for 15 s, 56 °C for 1 min
		R	GATCTCGCTCCTGGAAGATG	
